# Multisystem Inflammatory Syndrome of Adults (MIS-A) as Delayed Severe Presentation of SARS-CoV-2 Infection: A Description of Two Cases

**DOI:** 10.3390/jcm13226632

**Published:** 2024-11-05

**Authors:** Bernd Raffeiner, Marco Rojatti, Christian Tröbinger, Adriana Manuela Nailescu, Leonardo Pagani

**Affiliations:** 1 Department of Rheumatology, Central Hospital of Bolzano (SABES-ASDAA), Teaching Hospital of Paracelsus Medical University (PMU), 39100 Bolzano, Italy; 2 Department of Intensive Care Unit, Central Hospital of Bolzano (SABES-ASDAA), Teaching Hospital of Paracelsus Medical University (PMU), 39100 Bolzano, Italy; 3 Department of Internal Medicine, Central Hospital of Bolzano (SABES-ASDAA), Teaching Hospital of Paracelsus Medical University (PMU), 39100 Bolzano, Italy; 4 Department of Infectious Diseases, Central Hospital of Bolzano (SABES-ASDAA), Teaching Hospital of Paracelsus Medical University (PMU), 39100 Bolzano, Italy

**Keywords:** SARS-CoV2 infection, MIS-A, MIS-C, inflammation, heart involvement, thrombocytopenia, immunoglobulins

## Abstract

**Background:** SARS-CoV-2 infection can lead to a potentially life-threatening condition known as SARS-CoV-2-associated multisystem inflammatory syndrome in children (MIS-C), which differs from the severe lung disease and thrombotic complications commonly seen in adults. Recently, similar cases have been identified in adults, characterized by a clinical multisystem inflammatory syndrome referred to as MIS-A, which can emerge as a late and severe complication of SARS-CoV-2 infection. **Case Presentation:** We report two cases of MIS-A that were recently admitted to our hospital. Both patients developed a severe multisystem inflammatory syndrome despite experiencing only mild SARS-CoV-2 infection. Key clinical features in both cases included significant systemic inflammation, prominent cardiac involvement, and thrombocytopenia. Prior SARS-CoV-2 infection was confirmed through serological testing. Treatment protocols for MIS-C, including steroids and immunoglobulins, proved effective for both patients. **Conclusions:** Clinicians should remain vigilant for MIS-A in the context of ongoing SARS-CoV-2 infection worldwide. This infection, even when presenting with mild or no symptoms, can progress to a life-threatening hyperinflammatory syndrome with cardiac implications if not promptly recognized and treated.

## 1. Background

Since the emergence of SARS-CoV-2 in December 2019 in the Chinese city of Wuhan, the world has witnessed a range of dramatic consequences stemming from the viral pandemic. SARS-CoV-2 presents diverse clinical manifestations, ranging from mild infections to severe diseases characterized by high mortality rates. The virus directly damages multiple organs, particularly the lung parenchyma. In patients who develop severe disease, SARS-CoV-2 triggers an aberrant host immune response that can lead to acute respiratory distress syndrome (ARDS). The exacerbation of this immune response, along with immune dysregulation and the accumulation of cytokines (often referred to as a cytokine storm), results in extensive tissue damage, capillary leak, intravascular thrombus formation, and organ dysfunction [1]. Consequently, treatment strategies typically include oxygen therapy, ventilatory support, glucocorticoids [2], and low-molecular-weight heparin [3].

While children are generally less affected by severe respiratory disease, they can develop SARS-CoV-2-associated multisystem inflammatory syndrome (MIS-C), which is distinct from severe COVID-19. Most reported cases of MIS-C have shown positive serological testing for SARS-CoV-2, with less frequent positive RT-PCR results from nasopharyngeal swabs, suggesting that this syndrome may be post-infectious. MIS-C is characterized by a hyperinflammatory state (cytokine storm syndrome) that progresses to multiple organ involvement, particularly affecting cardiac myositis, coagulopathy, and macrophage activation syndrome (MAS). The mortality rate in US cohorts of MIS-C has been reported to be 2% [4].

In addition, cases of a similar multisystem inflammatory syndrome in adults (MIS-A) have been reported from the United Kingdom, the USA [5], Turkey [6], and Germany [7]. The diagnostic criteria for MIS-A have been established [5] and include the following five criteria: (1) a severe illness requiring hospitalization in a person aged ≥21 years; (2) a positive test result for current or previous SARS-CoV-2 infection (nucleic acid, antigen, or antibody) during admission or in the preceding 12 weeks; (3) severe dysfunction of one or more extrapulmonary organ systems (e.g., hypotension or shock, cardiac dysfunction, arterial or venous thrombosis or thromboembolism, or acute liver injury); (4) laboratory evidence of severe inflammation (e.g., elevated levels of CRP, ferritin, D-dimer, or interleukin-6); and (5) absence of severe respiratory illness (to exclude patients whose inflammation and organ dysfunction may be attributed merely to tissue hypoxia).

Despite Italy being severely impacted by the pandemic since March 2020, reports of MIS-A cases have been lacking. We present two cases recently admitted to our hospital.

## 2. Case Presentations

**Figure 1:**
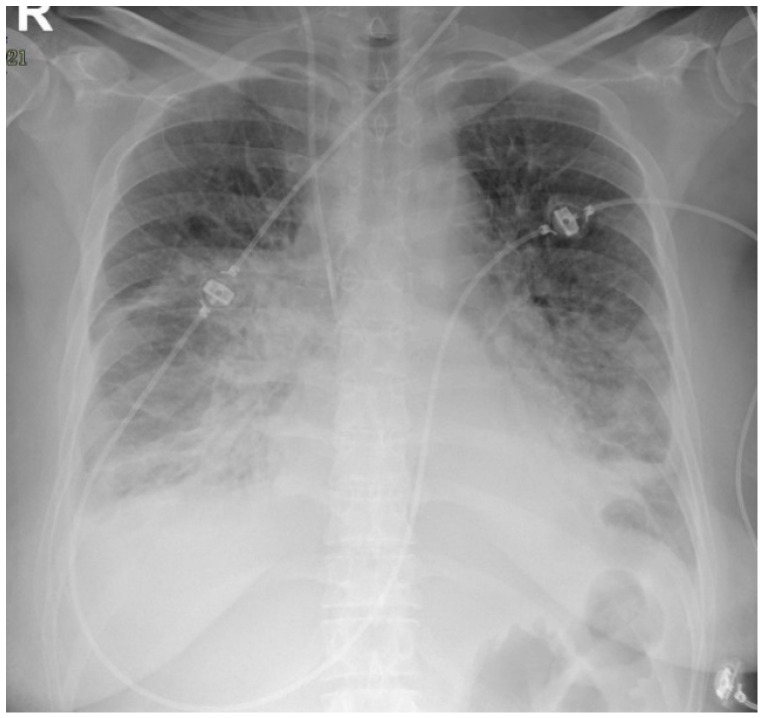
Case 1: showing lung edema and bilateral pleural effusion due to rapidly evolving MIS-A cardiac involvement.

First Case: A 47-year-old otherwise healthy woman was admitted for high fever, myalgia, abdominal pain, diarrhea, and malaise persisting for several days. Her nasopharyngeal swab for SARS-CoV-2 was negative, but she exhibited highly positive IgG levels against spike and capsid antigens, indicating a past infection. She had not reported any previous symptoms. Several weeks prior, some family members had tested positive for SARS-CoV-2. Blood tests revealed a systemic inflammatory response: leukocytosis (16,080/µL [4000–11,000/µL]), neutrophilia (15,310/µL [1500–8000/µL]), C-reactive protein (CRP) 33 mg/dL [<0.5 mg/dL], ferritin 770 ng/mL [13–150 ng/mL], procalcitonin 8.32 ng/mL [<0.05 ng/mL], IL-6 920 pg/mL [<7 pg/mL], hypoalbuminemia (2.2 g/dL [3.5–5.3 g/dL]), D-dimer elevation (7452 ng/mL [<300 ng/mL]), PT 1.6 INR [0.85–1.2], and moderate thrombocytopenia (75,000/µL [150,000–450,000/µL]). A CT scan excluded thoracoabdominal embolism and infectious foci. She rapidly developed severe hypotension and liver and kidney dysfunction resembling septic shock, requiring volume resuscitation, catecholamines, and hydrocortisone administration in the intensive care unit on the second day. On the third day, she developed atrial fibrillation, and a chest X-ray documented acute pulmonary edema and massive pleural effusions (Figure 1), necessitating pleural drainage. The pleural fluid was transudate. NT-proBNP levels increased from 478 pg/mL to 17,462 pg/mL [<125 pg/mL]. Echocardiography showed the left ventricle was at the upper limit of normal. She received CPAP ventilation, amiodarone, diuretics, and heparin infusion. Blood, urine, and stool cultures remained negative. Immunological testing results, including ANA, ENA, anti-DNA, rheumatoid factor, C4, ANCA, anti-phospholipid antibodies, and HIT antibodies (anti-PF4), were all negative; C3 was 71 mg/dL [90–180 mg/dL], IgG was 5.5 g/L [7.1 g/L], and lymphocytes were reduced (110/µL [1000–4800/µL]). We diagnosed MIS-A based on her clinical presentation, serological testing for COVID-19, and exclusion of alternative diagnoses. Treatment was initiated with methylprednisolone 40 mg IV twice daily and immunoglobulins at 1 g/kg for 2 days, following MIS-C protocols. The patient fully recovered, with heart function and examinations returning to normal within a few days. Glucocorticoids were tapered over three weeks.

**Figure 2:**
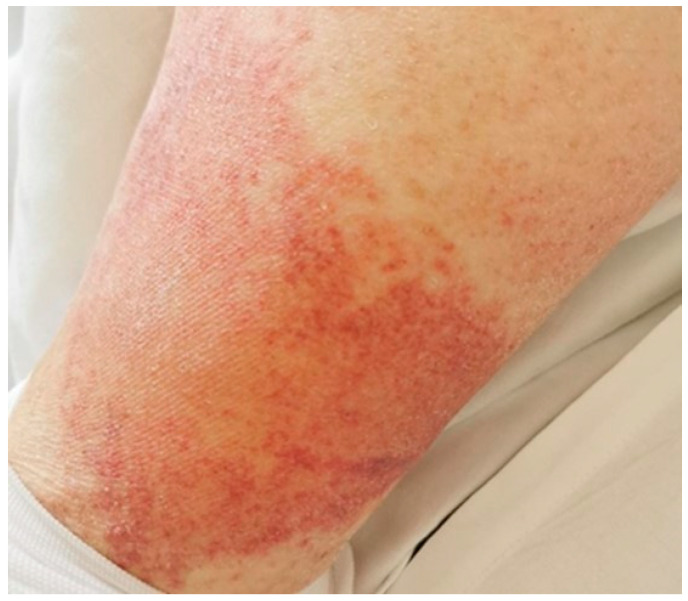
Case 2: purpuric rash appeared on patient’s lower extremities.

**Figure 3:**
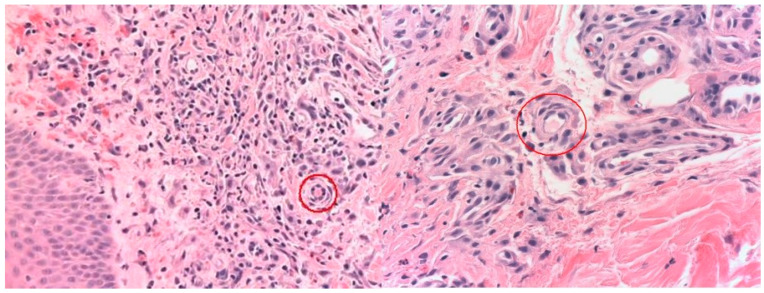
Case 2: skin biopsy with diffuse edema of the papillary derma and erythrocyte extravasation, capillary proliferation and microthrombi (red circle), interstitial and perivascular infiltration of lymphocytes, scattered eosinophils, and nuclear dust (HE ×20 and ×40).

Second Case: A 70-year-old man without any significant past medical history presented with fatigue and nonspecific myalgias over the past six weeks. He appeared febrile but otherwise unremarkable. Blood tests exhibited leukocytosis (18,060/µL [4000–11,000/µL]), neutrophilia (15,610/µL [1500–8000/µL]), lymphopenia (820/µL [1000–4800/µL]), CRP 32 mg/dL [<0.5 mg/dL], hypoproteinemia (5.2 g/dL [3.5–5.3 g/dL]), prolonged PT (1.5 INR [0.85–1.2]), and elevated D-dimer levels (4840 ng/mL [<300 ng/mL]). GOT was 142 U/L [<35 U/L] and GPT was 140 U/L [<35 U/L]. All SARS-CoV-2 swabs and other microbial cultures were negative, and several antibiotic treatments proved ineffective. Total-body CT scans revealed scant residual pulmonary infiltrates, which, along with highly positive serology for SARS-CoV-2, suggested a recently resolved asymptomatic COVID-19 infection. The patient did not report respiratory symptoms and did not require oxygen support. CT scans also revealed pleural effusions; NT-proBNP was measured at 478 pg/mL [<125 pg/mL], and echocardiography showed hyperkinetic left ventricular function. A PET scan excluded neoplasia and giant-cell arteritis. On the seventh day after admission, a purpuric rash appeared on the patient’s lower extremities (Figure 2), and platelet levels decreased from normal to 13,000/µL [150,000–450,000/µL]. The peripheral blood smear was negative for blasts. Immunoglobulin levels and lymphocyte typing were within normal ranges. Complement fractions were severely depleted, with C3 measured at 77 mg/dL [90–180 mg/dL] and C4 at <2 mg/dL [10–30 mg/dL]. Autoantibody tests, including ANA, ENA, anti-DNA, rheumatoid factor, myositis antibodies, ANCA, cryoglobulins, anti-phospholipid antibodies, and HIT antibodies, were all negative. A skin biopsy revealed diffuse edema in the papillary dermis, erythrocyte extravasation, capillary microthrombi, and interstitial and perivascular infiltration of inflammatory cells, including eosinophils and nuclear debris (Figure 3). Given the clinical presentation, recent SARS-CoV-2 infection, and the exclusion of other causes, we diagnosed MIS-A. Treatment with immunoglobulins and methylprednisolone infusions, following the regimen used in the first case, promptly resolved the patient’s fever, hyperinflammatory state, cardiac function, and platelet count. Glucocorticoids were gradually tapered and discontinued after three weeks.

## 3. Discussion

Approximately 50 cases of MIS-A, the adult equivalent of MIS-C, have been reported. Previous case series have detailed the clinical and laboratory characteristics and treatment modalities of, and developed diagnostic criteria for, MIS-A [5].

MIS-A is regarded as a post-infectious syndrome rather than an acute infection, as SARS-CoV-2 PCR results are predominantly negative while antibodies against SARS-CoV-2 are typically positive. There is a lack of clear evidence regarding the immune pathophysiology of this syndrome; however, an antibody-mediated immune response may be involved. In both of our cases, complement fractions were reduced, particularly in Case 2, which exhibited severe thrombocytopenia, indicating activation via the classical pathway linked to immune complex formation. No other specific alterations in the immune system were identified through conventional laboratory assessments, except for lymphopenia, which primarily affected T-helper and T-suppressor cells, while NK cells remained spared—this contrasts with findings in patients with macrophage activation syndrome (MAS).

In children, non-MIS-C and MIS-C SARS-CoV-2-associated illnesses are characterized by divergent immune signatures that are temporally distinct, implicating CD8 T cells in the clinical presentation and progression of MIS-C [8]. Some authors also speculate about the possibility of persistent infection occurring outside the upper respiratory tract. Additionally, more consistent mechanisms proposed include dysregulated immune responses, hyperinflammation, endothelial damage, and microthrombosis affecting the heart, liver, brain, kidneys, and gastrointestinal tract [9].

Both of our cases exhibited coagulopathy, attributed in part to liver damage but primarily due to thrombotic thrombocytopenia. In particular, the biopsy of the affected skin in our second case confirmed inflammatory infiltration, endothelial damage, and the formation of microthrombi, rather than simple petechial bleeding due to thrombocytopenia. Immunofluorescence analysis revealed no deposits of IgG, IgA, IgM, or complement. PCR testing for SARS-CoV-2 on skin specimens yielded negative results. The thrombocytopenia responded promptly to treatment with immunoglobulins and glucocorticoids. Additionally, both cases tested negative for HIT antibodies, given the thrombocytopenia that may have worsened after heparin administration in both cases, alongside recent findings of the development of anti-PF-4 antibodies and rare instances of thrombotic thrombocytopenic syndrome associated with viral vector vaccines [10]. Consequently, it appears that these two syndromes (MIS-A and vaccine-induced syndrome) do not share common immunological pathways.

Despite the presence of thrombotic thrombocytopenia and hyperinflammatory syndrome, the primary clinical feature in both patients was heart failure. Notably, the cardiac involvement in Case 1 resulted in severe hypotension, lung edema, and the onset of atrial fibrillation, along with pleural effusions that required drainage. Interestingly, echocardiographic assessments in both cases did not reveal significant structural or functional abnormalities, suggesting that cardiac dysfunction may have been underestimated. We hypothesize that the rapid development and subsequent resolution of cardiogenic pulmonary edema may be attributed to inflammatory microthrombotic involvement of the heart. In our cases, NT-proBNP emerged as a more reliable marker than troponin for assessing the severity of myocarditis.

In conclusion, MIS-A is a rare but emerging illness [11,12,13] characterized by microthrombotic thrombocytopenia that clinicians should be vigilant for, as it can evolve rapidly and atypically, leading to potentially life-threatening organ dysfunctions. Remarkably, neither patient exhibited COVID-19 symptoms during the acute phase, and it was only the manifestation of such severe features that revealed the recent infection.

## Data Availability

All clinical and laboratory data, imaging files, and histological specimens are available.

## Abbreviations

MIS-C	multisystem inflammatory syndrome in children
MIS-A	multisystem inflammatory syndrome in adults
MAS	macrophage activation syndrome
